# Experience-Dependent Plasticity and Modulation of Growth Regulatory Molecules at Central Synapses

**DOI:** 10.1371/journal.pone.0016666

**Published:** 2011-01-31

**Authors:** Simona Foscarin, Danilo Ponchione, Ermira Pajaj, Ketty Leto, Maciej Gawlak, Grzegorz M. Wilczynski, Ferdinando Rossi, Daniela Carulli

**Affiliations:** 1 Neuroscience Institute of Turin (NIT), Department of Neuroscience, University of Turin, Turin, Italy; 2 Neuroscience Institute of the Cavalieri-Ottolenghi Foundation (NICO), University of Turin, Orbassano, Turin, Italy; 3 Laboratory of Molecular and Systemic Neuromorphology, Nencki Institute of Experimental Biology, Warsaw, Poland; National Institute on Aging Intramural Research Program, United States of America

## Abstract

Structural remodeling or repair of neural circuits depends on the balance between intrinsic neuronal properties and regulatory cues present in the surrounding microenvironment. These processes are also influenced by experience, but it is still unclear how external stimuli modulate growth-regulatory mechanisms in the central nervous system. We asked whether environmental stimulation promotes neuronal plasticity by modifying the expression of growth-inhibitory molecules, specifically those of the extracellular matrix. We examined the effects of an enriched environment on neuritic remodeling and modulation of perineuronal nets in the deep cerebellar nuclei of adult mice. Perineuronal nets are meshworks of extracellular matrix that enwrap the neuronal perikaryon and restrict plasticity in the adult CNS. We found that exposure to an enriched environment induces significant morphological changes of Purkinje and precerebellar axon terminals in the cerebellar nuclei, accompanied by a conspicuous reduction of perineuronal nets. In the animals reared in an enriched environment, cerebellar nuclear neurons show decreased expression of mRNAs coding for key matrix components (as shown by real time PCR experiments), and enhanced activity of matrix degrading enzymes (matrix metalloproteinases 2 and 9), which was assessed by *in situ* zymography. Accordingly, we found that in mutant mice lacking a crucial perineuronal net component, cartilage link protein 1, perineuronal nets around cerebellar neurons are disrupted and plasticity of Purkinje cell terminal is enhanced. Moreover, all the effects of environmental stimulation are amplified if the afferent Purkinje axons are endowed with enhanced intrinsic growth capabilities, induced by overexpression of GAP-43. Our observations show that the maintenance and growth-inhibitory function of perineuronal nets are regulated by a dynamic interplay between pre- and postsynaptic neurons. External stimuli act on this interaction and shift the balance between synthesis and removal of matrix components in order to facilitate neuritic growth by locally dampening the activity of inhibitory cues.

## Introduction

Structural remodeling of neural circuits depends on the balance between intrinsic neuronal capabilities for neuritic growth and environmental signaling. During development, axon elongation and synaptogenesis are favored by the weak activity of inhibitory cues present in the CNS milieu. The end of ontogenetic processes, however, is marked by the appearance of regulatory molecules, including myelin-associated proteins and extracellular matrix (ECM) components, which restrict neuronal plasticity in order to stabilize specific connection patterns [1].

In the adult CNS, structural plasticity can be enhanced by manipulations that strengthen intrinsic neuronal properties or counteract extrinsic inhibitory mechanisms. Overexpression of growth-associated molecules in adult neurons stimulates neuritic outgrowth into unusual or prohibitive territories [2]–[4]. Similarly, procedures that neutralize environmental regulatory cues induce sprouting of adult axons [5]–[7], and allow functional modifications typical of developmental critical periods [8]–[11].

Physiological remodeling or repair processes are also strongly influenced by experience-dependent mechanisms, i.e. the interaction between neural circuits and the external world [12], [13]. Quite surprisingly, however, little is known about the influence exerted by external stimuli on the cellular/molecular mechanisms that regulate neuronal growth. Physical exercise or exposure to enriched environment (EE) stimulate neuritic growth and functional plasticity, and these effects are paralleled by changes in the expression of neurotrophins, neuronal growth genes and regulatory substances [14]–[17]. In addition, physiological stimulation by salt loading induces a decrease in the expression of ECM components in the hypothalamus [18]. Nevertheless, direct evidence that external stimulation promotes structural plasticity by locally modulating the activity of regulatory mechanisms is still lacking.

To address this issue, we asked whether EE induces synapse remodeling and modulation of perineuronal nets (PNN) in the adult deep cerebellar nuclei (DCN). PNNs are accumulations of ECM that enwrap the perikaryon of defined neuronal populations, including DCN neurons [19]. Their formation at the end of development has been related to the closure of critical periods and the restriction of plasticity [8], [11]. EE enhances learning and memory [12], promotes experience-dependent plasticity in the adult visual system [17], [20], and improves compensatory processes in the damaged CNS [12], [21]. Our observations indicate that the PNN structure is maintained through a dynamic interaction between DCN neurons and their main afferents, the axons of Purkinje cells (PC). EE shifts the balance of this interaction to reduce growth-inhibitory components. This effect is further amplified if PC axons are endowed with enhanced intrinsic growth properties, highlighting a complex triadic interplay between neuronal genes, inhibitory molecules and external stimuli in the control of CNS plasticity.

## Results

### EE induces structural plasticity of PC and glutamatergic axons in the cerebellar nuclei

**Figure 1:**
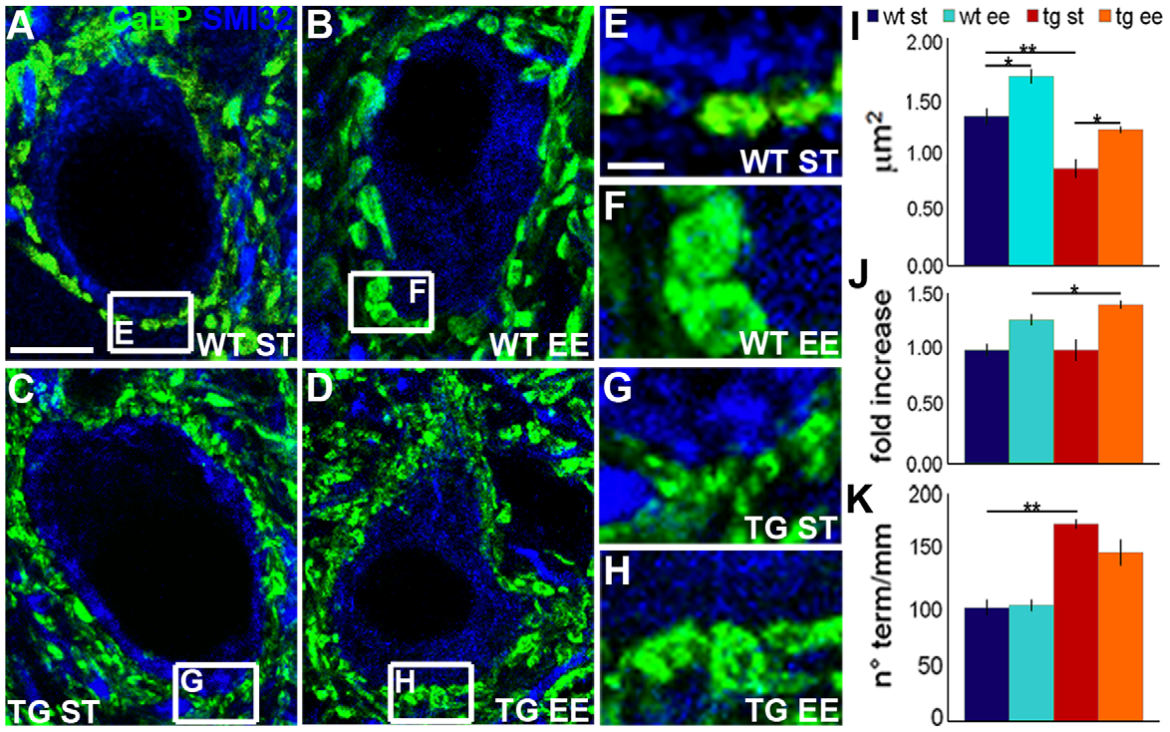
Size changes of PC axon terminals following exposure to EE. (A-H) DCN neurons, visualized by SMI32 antibodies (blue), are contacted by PC axon terminals, stained by anti-calbindin antibodies (green). In both wild-type (A,B,E,F) and transgenic mice (C,D,G,H), PC terminals are enlarged after EE (B,F,D,H). (I) Size of PC axon boutons in the different experimental conditions (One Way Anova; N = 4 wild-type and transgenic ST; N = 10 wild-type EE and 9 transgenic EE mice). (J) Relative size increase of PC terminals induced by EE in wild-type and transgenic mice (Student's t-test; N = 10 wild-type, N = 9 transgenic mice). (K) Number of PC terminals/mm of post-synaptic membrane of DCN neurons (One Way Anova; N = 5 mice/experimental condition). *P<0.05; **P<0.01. Scale bars: 10 µm (A–D), 2 µm (E–H). WT: wild-type; TG: transgenic; ST: standard condition; EE: enriched condition; CaBP: calbindin; SMI32: neurofilament-H non-phosphorylated.

The large projection neurons of the DCN receive strong innervation from GABAergic PC axons on the perikaryon, whereas excitatory synapses of glutamatergic mossy fibers and olivocerebellar axons impinge upon the dendrites [22]. To ask whether external stimulation induces structural plasticity in the DCN, we compared morphometric measurements of synaptic terminals of mice reared in standard conditions (ST) or exposed to EE for one month. In the cerebella of mice exposed to EE, PC boutons contacting DCN neurons were consistently larger than those from animals reared in ST conditions (**Fig. 1A,B,E,F,I**; ST = 1.31 µm^2^±0.07; EE = 1.66 µm^2^±0.06; One Way Anova: *P* = 0.006; statistical parameters are reported in the figure legends). To assess whether such EE-induced changes of PC terminals were influenced by the intrinsic growth properties of the parent neurons, we repeated the same experiments with transgenic mice in which the growth-associated protein GAP-43 is overexpressed under control of the PC specific promoter L7 [3]. Although PC terminals of L7/GAP-43 mice reared in ST conditions were smaller than those of their wild-type counterparts (**Fig. 1A,C,E,G,I**; L7/GAP-43 ST = 0.85 µm^2^±0.08; One Way Anova: *P* = 0.003), they were considerably enlarged after exposure to EE (**Fig. 1C,D,G,H,I**; L7/GAP-43 EE = 1.19 µm^2^±0.03; One Way Anova: *P* = 0.009), and this size modification was significantly larger than that observed in wild-type animals (**Fig. 1J**; 27% increase in wild-type, 40% increase in transgenic mice; Student's t-test: *P* = 0.046).

Next, we examined whether the number of PC terminals/mm of postsynaptic membrane of DCN neurons was also affected by EE. In this case, we did not find any significant difference between EE and ST conditions for both wild-type (**Fig. 1K**; ST = 99.79±7.08; EE = 101.93±5.12; One Way Anova: *P* = 1.000) and L7/GAP-43 mice (**Fig. 1K**; ST = 173.24±4.22; EE = 148.22±11.35; One Way Anova: *P* = 0.184), although the values calculated in the latter mice were 73% higher than in wild-type mice (**Fig. 1K**; One Way Anova: *P*<0.001). The perikaryal size of DCN neurons (as well as of PCs) were similar in all experimental conditions (**Fig. S1**), thus indicating that EE caused an enlargement of PC boutons but did not change their number.

**Figure 2:**
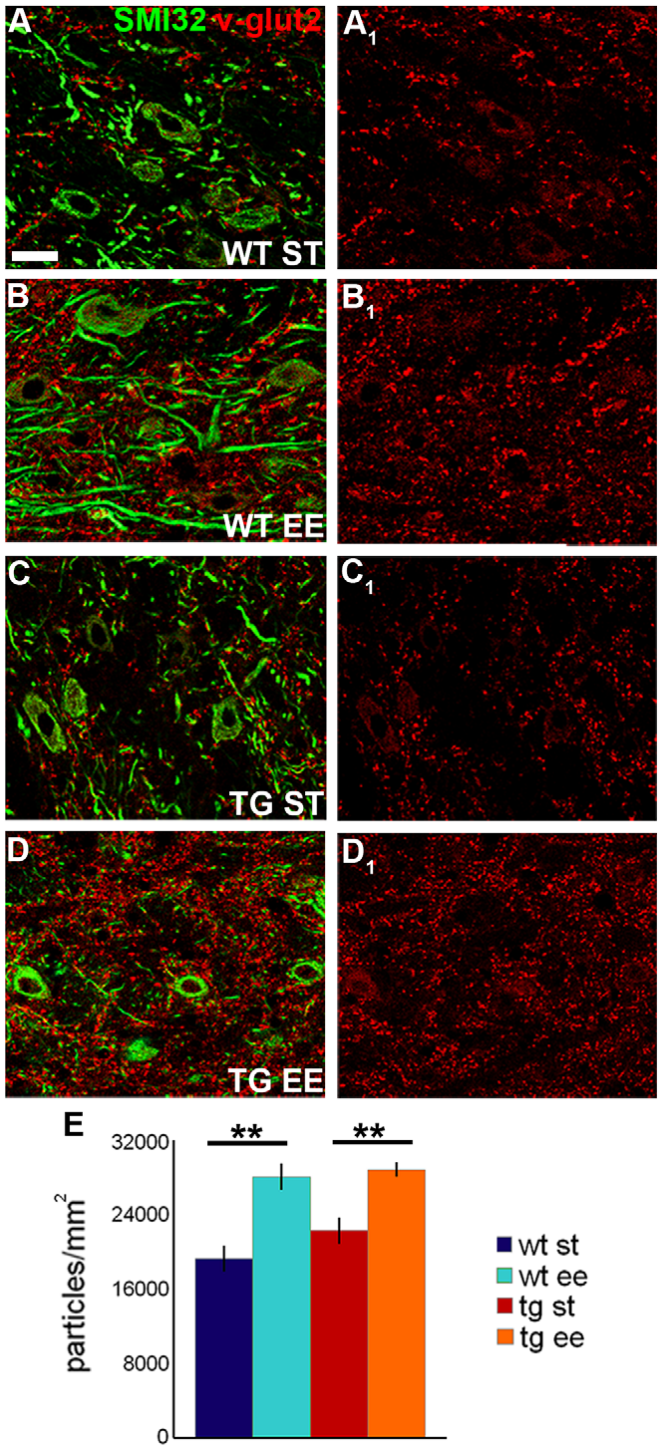
Changes of glutamatergic terminal density after EE. (A–D_1_) The micrographs show the distribution pattern of excitatory mossy fiber and olivocerebellar axon terminals, stained by anti-v-glut2 antibodies (red), in the DCN. DCN neurons are visualized by SMI32 antibodies (green). (E) Quantification of the density of glutamatergic terminals in the DCN in the different experimental conditions (One Way Anova; N = 4 ST and EE wild-type mice, N = 7 ST and EE transgenic mice). **P<0.01. Scale bar: 20 µm. WT: wild-type; TG: transgenic; ST: standard condition; EE: enriched condition; SMI32: neurofilament-H non-phosphorylated; v-glut2: second vesicular glutamate transporter.

To ask whether EE induced modifications of mossy fibers and olivocerebellar axons, we examined boutons labeled by anti-v-glut2 antibodies. Since these afferents impinge upon the dendrites of DCN neurons, which are not clearly visualized by immunocytochemistry, we estimated the number of terminals per mm^2^ of DCN. The density of glutamatergic terminals was consistently higher in EE than in ST conditions both in wild-type (**Fig. 2A,B,E**; ST = 19263.99±1403.57; EE = 28077.15±1434.58; One Way Anova: *P* = 0.003) and in L7/GAP-43 mice (**Fig. 2C,D,E**; ST = 22274.76±1369.52; EE = 28839.63±758.14; One Way Anova: *P* = 0.003). In this case, however, we found no difference between wild-type and transgenic animals in either environmental condition. Therefore, EE increases the density of glutamatergic boutons in the DCN, but this effect is not influenced by the intrinsic growth properties of PCs.

### The number and staining intensity of PNNs in the DCN are decreased after EE

**Figure 3:**
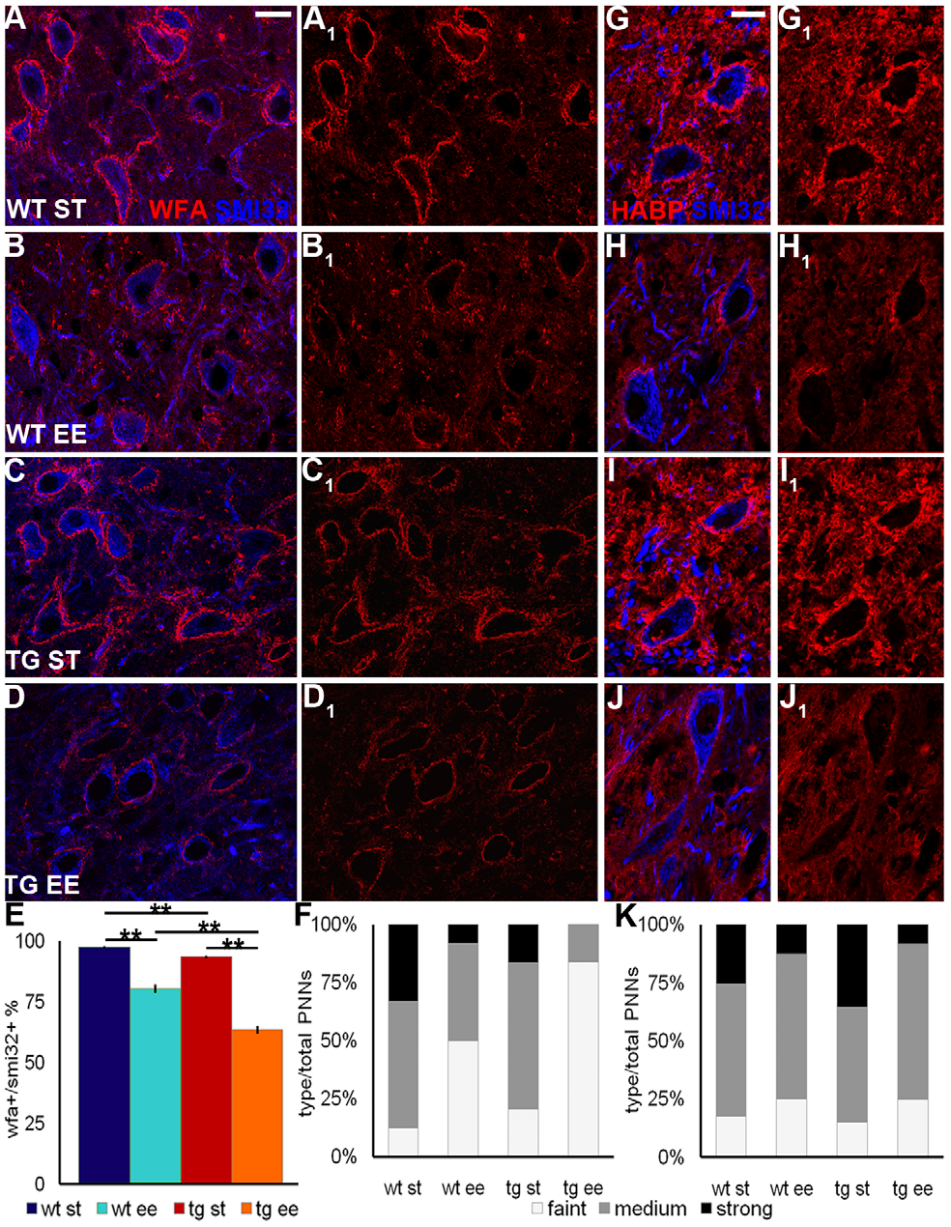
PNNs decrease after EE. (A–D_1_) PNNs, stained by WFA (red), enwrap DCN projection neurons, labeled by SMI32 antibodies (blue). (E) Percentage of SMI32-positive neurons bearing a WFA-positive net in the different experimental conditions (One Way Anova; N = 5 mice/experimental condition). (F) Frequency distribution of PNNs subdivided in three categories according to their WFA staining intensity (see methods; χ^2^-test: 343.04 with 6 DF). (g–j_1_) HABP staining of PNNs (red) around SMI32-positive neurons (blue). (K) Frequency distribution of HABP-positive nets subdivided in three categories according to their staining intensity (χ^2^-test: 38.77 with 6 DF). **P<0.01. Scale bar: 20 µm. WT: wild-type; TG: transgenic; ST: standard condition; EE: enriched condition; SMI32: neurofilament-H non-phosphorylated; WFA: *Wisteria floribunda* agglutinin; HABP: hyaluronan binding protein.

DCN projection neurons bear prominent PNNs composed of chondroitin sulfate proteoglycans (CSPGs), including aggrecan, versican, neurocan, brevican and phosphacan, hyaluronan and link proteins [19]. Given the well-established growth-inhibitory properties of these molecules, we asked whether plastic changes of PC terminals and precerebellar axons were accompanied by changes of PNNs in the DCN. First, we evaluated the number of DCN neurons that bore a PNN, visualized by *Wisteria floribunda* agglutinin (WFA) staining. In ST conditions, the fraction of DCN neurons enwrapped by a net was 97.57% (±0.51) in wild-type and 93.60% (±0.59) in L7/GAP-43 mice, and this slight difference resulted statistically significant (**Fig. 3A,C,E**; One Way Anova: *P* = 0.007). Following exposure to EE, these values were decreased to 80.45% (±1.77) and 63.53% (±1.63), respectively (**Fig. 3B,D,E**). Both values were significantly different than those observed in the relevant mouse strain maintained in ST conditions (One Way Anova: *P*<0.001 for both wild-type and L7/GAP-43 mice). In addition, the EE-induced change of PNN number was significantly larger in transgenic than in wild-type mice (One Way Anova: *P*<0.001; **Fig. 3E**). The same results were obtained when the three DCN (medial, interpositus and lateral nucleus) were evaluated individually (**Fig. S2**), showing that the effect of environmental stimulation was widespread.

To further characterize EE-dependent changes in PNN structure, we divided the sampled nets in three categories according to the intensity of WFA staining. Analysis of the relative frequencies of these categories in ST conditions showed that PNNs of L7/GAP-43 mice are significantly fainter than their counterparts of wild-type mice (χ^2^-test: *P*<0.001. **Fig. 3F**). After EE, the frequency of strongly stained PNNs decreased in both wild-type and transgenic animals, while the fraction of faintly stained PNNs increased in both groups (χ^2^-test: *P*<0.001; **Fig. 3F**). Again, the effect was more pronounced in L7/GAP-43 mice (χ^2^-test: *P*<0.001).

Exposure to EE also induced a decrease in the staining intensity of hyaluronan, the backbone of all organized matrices, which was visualized by biotinylated hyaluronan binding protein (HABP) binding (**Fig. 3G–J** **_1_**). Quantitative evaluation revealed a significant reduction of labeling in the EE condition for both wild-type and transgenic mice (χ^2^-test between ST and EE-mice: *P*<0.05; **Fig. 3K**).

### The absence of cartilage Link Protein 1 leads to increased size of Purkinje axon terminals

**Figure 4:**
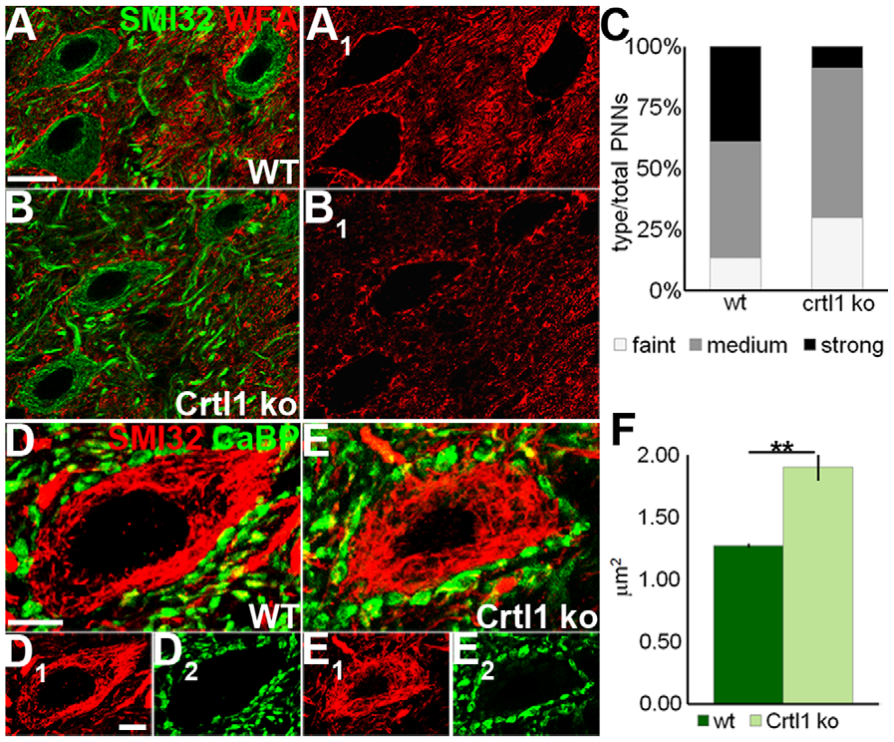
Morphology of PNN and PC terminals in cartilage LP1 knockout mice. (A–B_1_) show the appearance of WFA-positive nets (red), surrounding SMI32-positive DCN neurons (green) in wild-type (A,A_1_) and cartilage LP1 knockout mice (B,B_1_). (C) Frequency distribution of PNNs subdivided in three categories according to their WFA staining intensity (χ^2^-test: 52.26 with 2 DF). (D–E_2_) Morphology of PC terminals (green) impinging upon DCN neurons (red) in wild-type (D-D_2_) and cartilage LP1 knockout mice (E–E_2_). (F) Size of PC axon terminals in the two strains (Student's t-test; N = 3 wild-type, N = 4 knockout mice). **P<0.01. Scale bars: 20 µm in A–B_1_, 10 µm in D–E_2_. WT: wild-type; Crtl1 KO: cartilage LP1 knockout; WFA: *Wisteria floribunda* agglutinin; SMI32: neurofilament-H non-phosphorylated; CaBP: calbindin.

Cartilage Link Protein 1 (LP1) is a crucial protein for the formation and maintenance of PNN structure. In mutant mice lacking cartilage LP1 in the CNS [23], PNNs are attenuated in several sites and plasticity is enhanced in the adult visual cortex and in the denervated cuneate nucleus [11]. Thus, we asked whether the presence of defective PNNs in the intact cerebellum is sufficient to induce changes in PC axon terminals in the absence of enhanced environmental stimulation. In the DCN of cartilage LP1 knockout animals, WFA staining around neurons was attenuated (**Fig. 4A–C**; χ^2^-test: *P*<0.001), whereas PC terminals were significantly larger than those of wild-type mice (wild-type  = 1.27 µm^2^±0.02; knockout  = 1.90 µm^2^±0.11; Student's t-test: *P* = 0.005; **Fig. 4D–F**), suggesting that reduced inhibitory signaling conveyed by poorly developed PNNs may allow PC plasticity.

### The synthesis of key PNN components is decreased after EE

The EE-dependent reduction of PNNs may be due to a decrease in the synthesis of ECM molecules or to an increase in their degradation. To highlight changes in the synthesis of PNN components, we compared the expression of mRNA coding for key PNN molecules, including cartilage LP1, aggrecan, and hyaluronan synthase (HAS), in the DCN of mice exposed to EE or ST conditions. By *in situ* hybridization, we found that these molecules are synthesized by neurons of the mouse DCN (**Fig. S3**), consistently with previous observations in the rat cerebellum [19].

**Figure 5:**
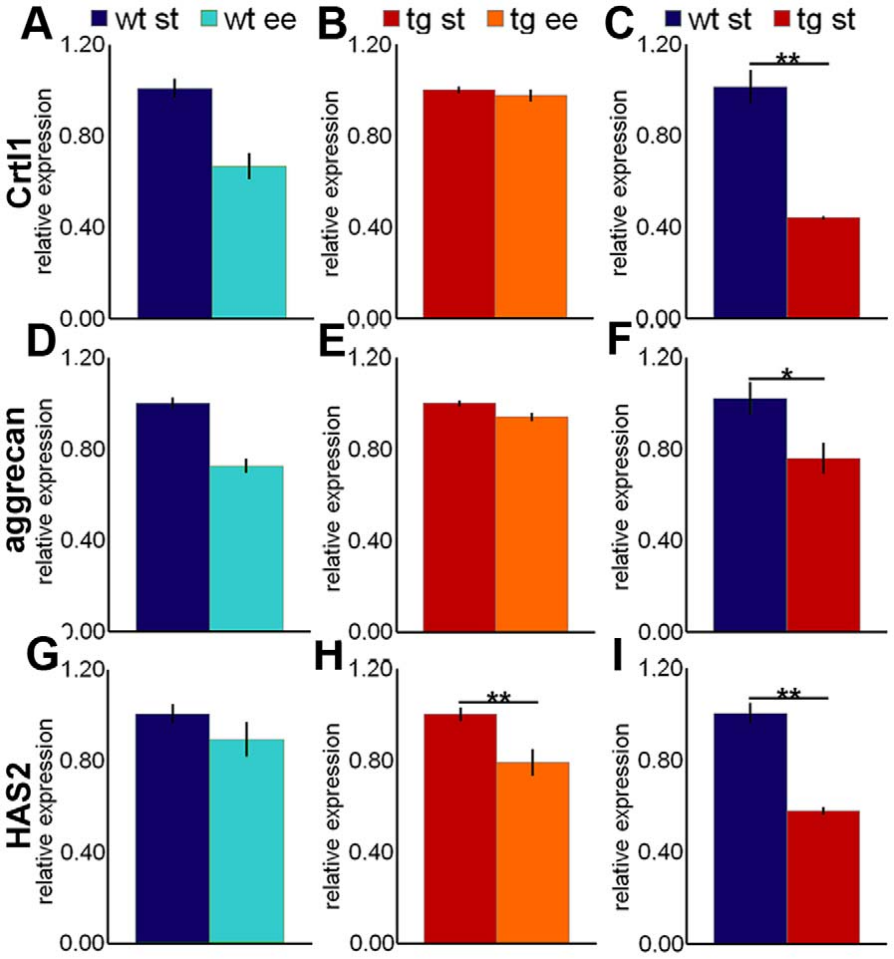
Expression of mRNA for PNN components in the DCN following EE. (A–I) Real-Time PCR experiments on DCN extracts show the level of expression of mRNA for cartilage LP1 (A–C: Student's t-test, N = 6–10/experimental condition), aggrecan (D–F: Student's t-test, N = 9/experimental condition), and HAS2 (G–I: Student's t-test, N = 6/experimental condition) in wild-type (A,D,G) or L7/GAP-43 mice (B,E,H) reared in ST conditions or EE. (C,F,I) compare basal level of the three molecules in the two mouse strains in ST conditions. *P<0.05; **P<0.01. Crtl1: cartilage LP1; HAS2: hyaluronan synthase 2.

Real-time PCR experiments on wild-type DCN showed a 33% decrease in cartilage LP1 mRNA after EE (ST = 1.01±0.04; EE = 0.67±0.06; Student's t-test: *P*<0.001; **Fig. 5A**). On the contrary, in L7/GAP-43 mice, cartilage LP1 mRNA levels were not changed after EE (ST = 1.00±0.02; EE = 0.98±0.03; Student's t-test: *P* = 0.437; **Fig. 5B**). Interestingly, however, comparison of wild-type and transgenic mice in ST conditions showed that the latter have only 45% of the mRNA levels of cartilage LP1 (wild-type = 1.01±0.18; transgenic = 0.44±0.02; Student's t-test: *P* = 0.002; **Fig. 5C**).

Aggrecan mRNA levels showed a 27% decrease after EE in wild-type mice (ST = 1.00±0.02; EE = 0.73±0.03; Student's t-test: *P*<0.001; **Fig. 5D**), and a 6% decrease in transgenic mice (ST = 1.00±0.01; EE = 0.94±0.02; Student's t-test: *P* = 0.022; **Fig. 5E**). When we compared the two strains in ST conditions, we found that the levels of aggrecan mRNA in transgenic DCN were 25% lower than in wild-type cerebella (wild-type = 1.02±0.07; transgenic = 0.76±0.07; Student's t-test: *P* = 0.018; **Fig. 5F**).

Three isoforms of HASs have been identified in mammals, HAS1, 2 and 3 [24]. In wild-type mice exposed to EE, HAS2 mRNA showed a slight tendency to decrease that was not statistically significant (ST = 1.01±0.04; EE = 0.89±0.08; Student's t-test: *P* = 0.234; **Fig. 5G**). On the contrary, in the EE-reared L7/GAP-43 mice, this mRNA was significantly decreased by 21% (ST = 1.00±0.03; EE = 0.79±0.06; Student's t-test: *P* = 0.009; **Fig. 5H**). Moreover, comparison of wild-type and transgenic mice in ST conditions showed that the latter expressed 42% less HAS2 mRNA (wild-type = 1.01±0.04; transgenic = 0.58±0.02; Student's t-test: *P*<0.001; **Fig. 5I**). Concerning the other HAS isoforms, HAS1 mRNA did not change after EE (not shown), whereas HAS3 mRNA was not detectable in the DCN.

### EE modifies the activity of matrix metalloproteinases

**Figure 6:**
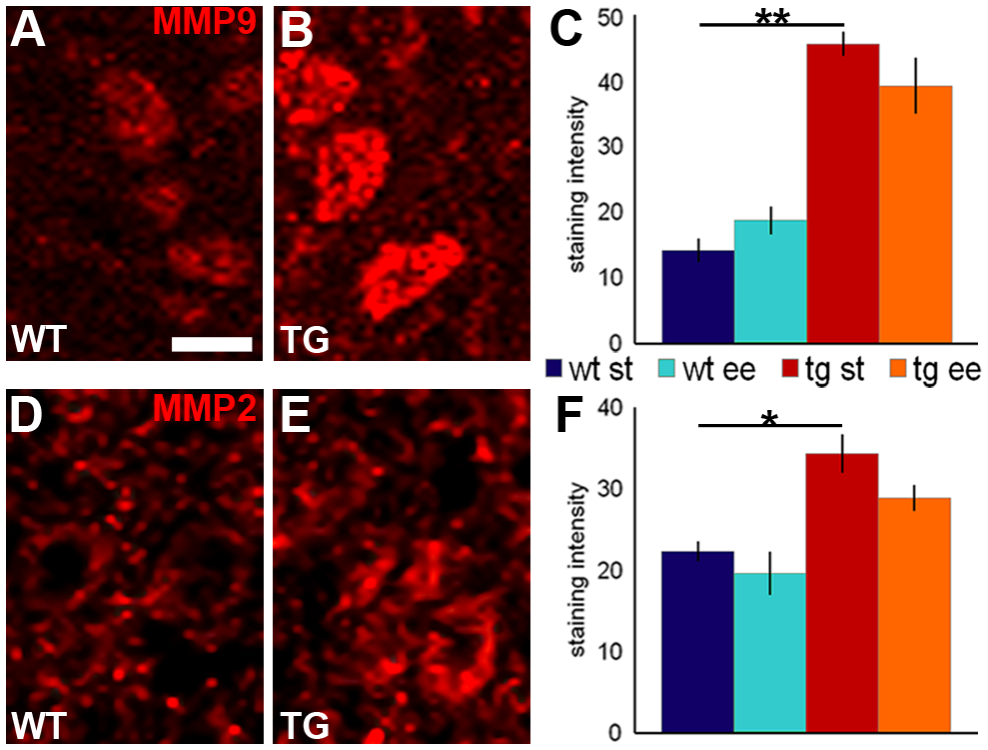
Immunolabeling for MMP9 and MMP2 in the DCN. (A,B,D,E) Immunohistochemistry for MMP9 (A,B) and for MMP2 (D,E) in wild-type (A,D) and transgenic (B,E) animals in ST conditions. (C,F) Quantification of the staining intensity for MMP9 (C; One Way Anova; N = 3 mice/experimental condition) and MMP2 (F; One Way Anova; N = 3 mice/experimental condition) in the four experimental conditions. *P<0.05; **P<0.01. Scale bar: 20 µm. WT: wild-type; TG: transgenic; MMP9: matrix metalloproteinase 9; MMP2: matrix metalloproteinase 2.

Matrix metalloproteinases (MMPs) are proteolytic enzymes that degrade ECM molecules, including CSPGs [25], [26]. To assess whether MMPs play a role in the PNN reduction in the DCN of EE-reared mice, we checked protein expression levels and enzymatic activity of two MMPs that are expressed in the cerebellum, MMP2 and MMP9 (gelatinases) [27]. MMP9 immunoreactivity was detected in the soma and dendrites of PCs (**Fig. S4A**), as well as in DCN projection neurons, interneurons and astrocytes (**Fig. S4B–D**). MMP2 immunoreactivity was also present in DCN neurons and glia, but not in PCs. For both proteins, staining intensity in DCN neurons was constitutively stronger in L7/GAP-43 than in wild-type cerebella (MMP9: transgenic = 45.58±1.85, wild-type = 14.14±1.82; One Way Anova: *P*<0.001; **Fig. 6A–C**. MMP2: transgenic = 34.30±2.37, wild-type = 22.32±1.24; One Way Anova: *P* = 0.019; **Fig. 6D–F**), but it was not modified after exposure to EE (One Way Anova: *P* > 0.05; **Fig. 6C,F**). Thus, the intensity of immunolabeling for gelatinases in DCN neurons is affected by the overexpression of GAP-43 in PCs, but does not change after EE.

**Figure 7:**
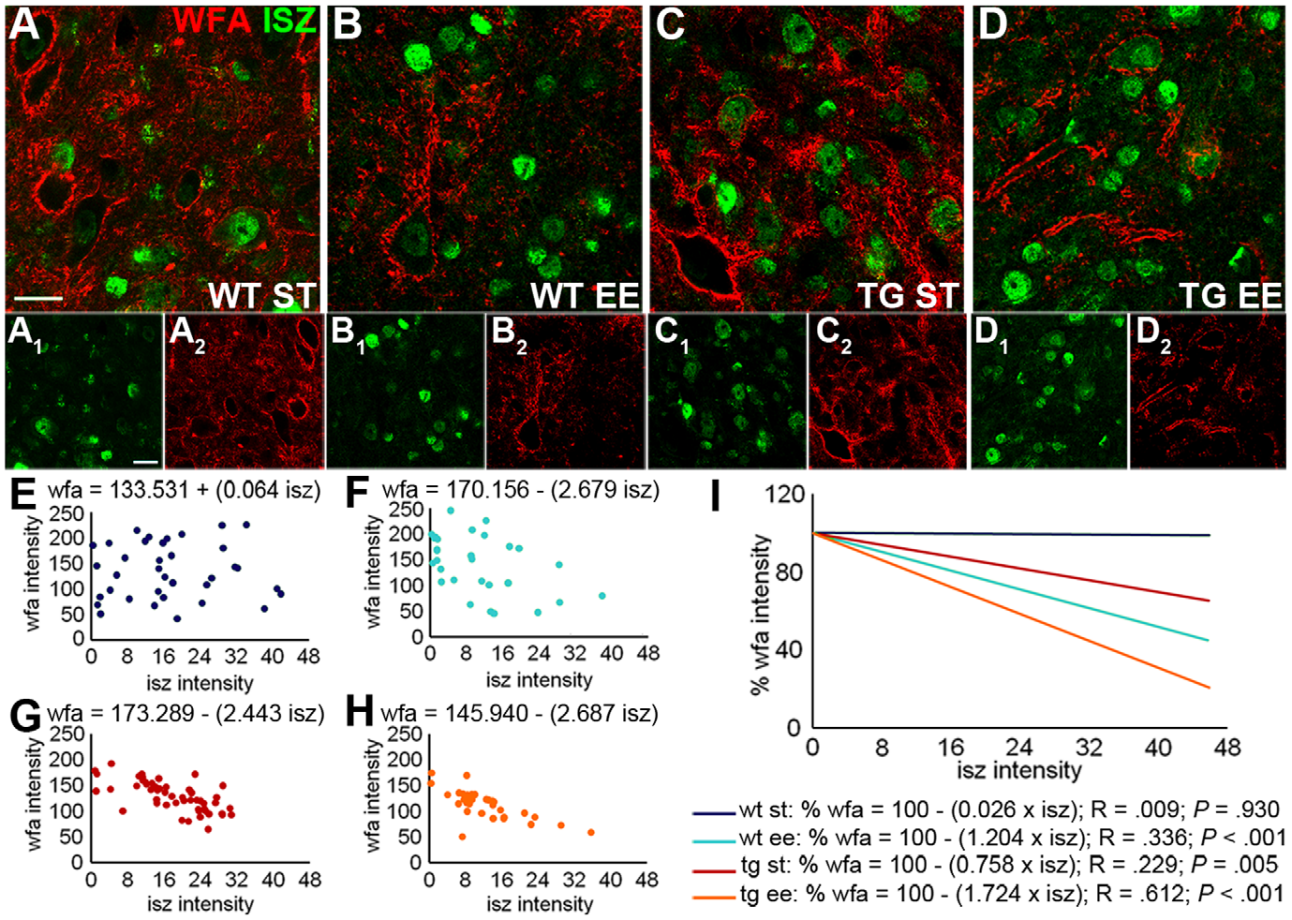
Relationship between MMP activity and PNN intensity in the DCN. (A–D_2_) show the ISZ staining (green) of DCN neurons and WFA labeling of PNNs (red) in the different experimental conditions. (E–H) Representative examples of the relationship between ISZ and WFA staining intensity in individual neurons (each plot reports data from a single animal for each group). (I) Regression lines calculated for each experimental group (N: wild-type ST = 106, wild-type EE = 167, transgenic ST = 148, transgenic EE = 176): the different regression lines have been plotted with the same intercept value (100), in order to compare the obtained slopes (One Way Anova on angular coefficients). Regression parameters are reported below the graph. Scale bars: 20 µm. WT: wild-type; TG: transgenic; ST: standard condition; EE: enriched condition; ISZ: *In situ* zymography; WFA: *Wisteria floribunda* agglutinin.

MMPs are produced as inactive enzymes and their activation requires the cleavage of a pro-peptide domain. Therefore, the identification of MMPs protein expression does not necessarily provide information about their enzymatic activity. To examine whether the expression of MMPs in the DCN corresponds to enzymatically active molecules, we performed high resolution *in situ* zymography (ISZ), involving the conversion of a non-fluorescent substrate (gelatin) into a fluorescent product where gelatinolytic activity is present [28]. Enzymatic activity was observed in PC bodies and dendrites (**Fig. S5A–D**) and in DCN neurons (**Fig. 7A–D**), whereas no signal was detected in slices treated with an MMPs inhibitor as a negative control (see **Methods** and **Fig. S6**). Quantitative analysis of ISZ labeling in the cerebellar cortex showed that EE tended to increase both the frequency of labeled PCs (**Fig. S5E**; wild-type: ST = 74.29%±2.18, EE = 87.06%±2.91; One Way Anova: *P* = 0.182. Transgenic: ST = 79.96%±5.81, EE = 93.76% ±1.89; One Way Anova: *P* = 0.05) and the intensity of ISZ signal (**Fig. S5F**). The latter effect was more pronounced in transgenic mice, in which the distribution of the categories of ISZ intensity was significantly shifted toward the stronger staining after EE (**Fig. S5C,D,F**; χ^2^-test: *P*<0.001 between ST and EE condition).

DCN neurons showed uneven levels of ISZ labeling (**Fig. 7A–D**). Therefore, we asked whether enzymatic activity in individual neurons was related to the intensity of WFA staining of the surrounding PNN. The resulting plots revealed an inverse relationship between ISZ and WFA intensity after EE (examples from representative animals are illustrated in **Fig. 7E–H**), as shown by an increase in both the angular and the regression coefficients of the calculated regression lines (**Fig. 7I**; angular coefficients in wild-type mice: ST = −0.249±0.36, EE = −1.204±0.26; One Way Anova: *P* = 0.039). As for many of the parameters considered in this study, in L7/GAP-43 mice the relationship was stronger than in wild-type mice, and EE induced a strengthening of this relationship, as seen by comparing the angular and regression coefficients of the regression lines (**Fig. 7I**; angular coefficients: ST = −0.758±0.27, EE = −1.724±0.17; One Way Anova: *P* = 0.002). On the whole, EE induced a strong reduction of WFA staining in PNNs that was related to the level of MMP activity in the corresponding neurons and this effect was enhanced in L7/GAP-43 mice.

### PNN modifications in partially-deafferented DCN

**Figure 8:**
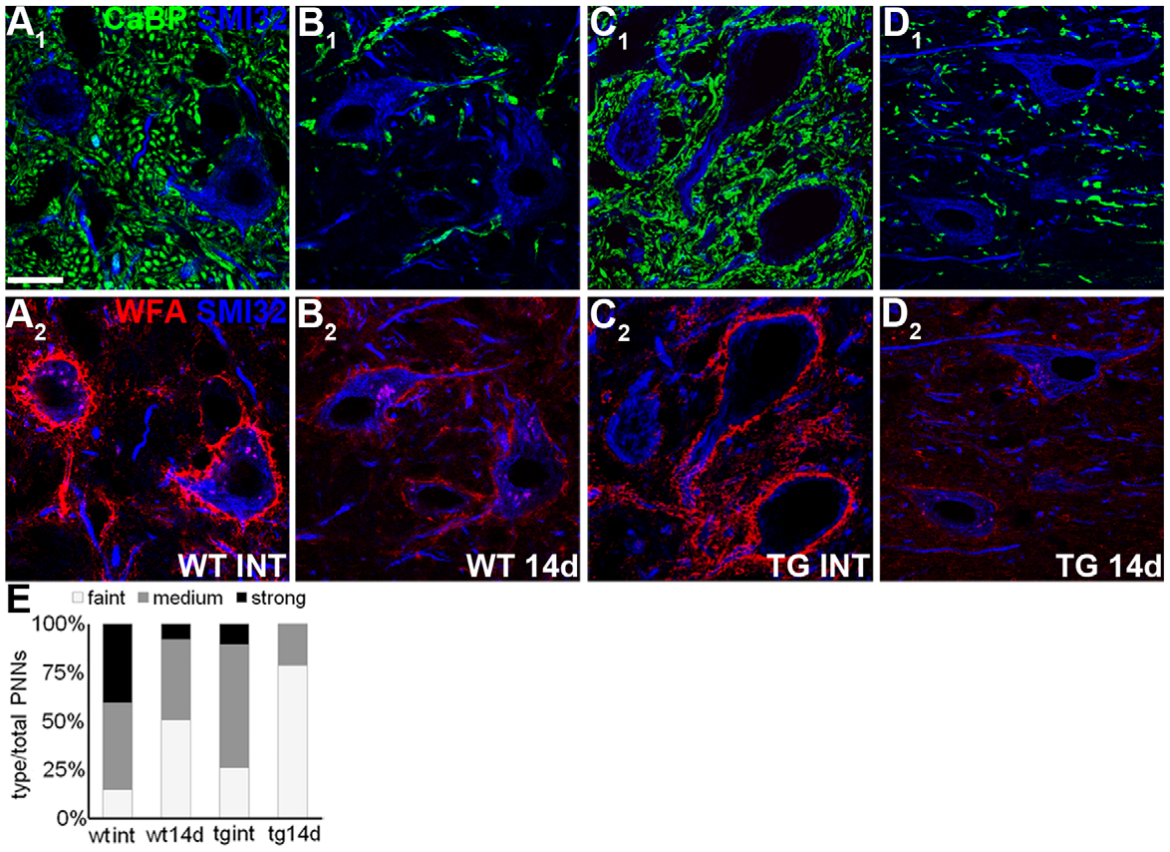
Morphology of PNNs in partially denervated DCN. (A_1_–D_2_) DCN neurons in intact and denervated wild-type and L7/GAP-43 mice (14 days after propidium iodide injection): SMI32-positive DCN neurons are visualized in blue, calbindin-immunolabeled PC terminals are green (A_1_–D_1_), and WFA stained PNNs are red (A_2_–D_2_). (E) Frequency distribution of PNNs subdivided in three categories according to their WFA staining intensity (χ^2^-test: 188.1 with 6 DF). Scale bar: 20 µm. WT: wild-type; TG: transgenic; ST: standard condition; EE: enriched condition; CaBP: calbindin; SMI32: neurofilament-H non-phosphorylated; WFA: *Wisteria floribunda* agglutinin.

The results obtained with L7/GAP-43 mice indicate that afferent axons, and notably those of PCs, contribute to regulate the structure and intensity of PNNs around DCN neurons. Thus, to further characterize the role of the presynaptic input in maintaining the structure of PNNs, we performed selective ablation of PCs by injecting propidium iodide in the cerebellar cortex [29]. Propidium iodide selectively kills PCs, while sparing all the other cerebellar cells (**Fig. S7A,B**). Given the topographic distribution of PC axons projecting to the DCN [22], a single injection of propidium iodide into the vermal lobules leads to a well circumscribed area of denervation in the medial nucleus (**Fig. S7A,C,D** and **Fig. 8B** **_1_,D_1_**). The intensity of WFA staining of individual PNN was assessed in such deafferented region 14 days after PC ablation (**Fig. 8A** **_2_–D_2_**). As shown in **Fig. 8**, in both wild-type and L7/GAP-43 mice, the loss of innervation induced a reduction of PNNs staining intensity, which was more pronounced in transgenic mice (the frequency distribution of strong, medium and faint PNNs was significantly different in all cases; χ^2^-test: *P*<0.001; **Fig. 8E**).

## Discussion

Different conditions that elicit functional plasticity, such as exposure to EE in the visual cortex [17] or salt loading in the supraoptic nucleus of the hypothalamus [30], [18], are accompanied by attenuation of PNNs. Here, we examined the effects of EE in the DCN to provide evidence that the changes of ECM components are actually related to local neuritic remodeling, and to elucidate the mechanism by which external stimuli modulate growth control in the adult CNS. Our results show that enhanced environmental stimulation induces significant morphological changes of PC and precerebellar axons, associated with a conspicuous reduction of PNNs.

Exposure to EE reduces the synthesis of key PNN components and, simultaneously, enhances their enzymatic degradation. PNN maintenance is determined by the interaction between the postsynaptic DCN neurons and their main presynaptic input, the PC axons. Most interestingly, the effects of EE are significantly potentiated when PC axons are endowed with enhanced intrinsic growth capabilities. Together, these observations indicate that the expression and function of growth-inhibitory cues are regulated by a dynamic interplay between pre- and postsynaptic neurons, driven by activation patterns induced by external stimuli. In this way, interaction with the external world modulates growth-regulatory mechanisms to create permissive conditions for local neuritic remodeling.

### EE-dependent synaptic remodeling in the DCN

Exposure to EE induced the enlargement of PC boutons, whose number was unchanged, and increased the density of glutamatergic terminals. These observations, that add on previously reported EE-dependent structural remodeling in the cerebellar cortex [31], [32], are consistent with a previous study, in which the number of PC synapses was not modified after complex motor learning [33]. On the other hand, enlarged boutons have been observed in conditions of PC hyperactivity [34], that may lead to reduced inhibitory influence on DCN neurons [35]. Complex functional interactions between inhibitory PC input and excitatory extracerebellar afferents to DCN neurons are required to induce potentiation of the latter synapses, which has been related to motor learning [36]. On the whole, the structural changes observed in the mice exposed to EE are suggestive of a weakening of PC inhibitory control, accompanied by strengthened excitatory drive of DCN neurons, thus facilitating cerebellar output.

### EE-dependent synaptic remodeling is associated with changes of PNN structure

The EE-induced reduction of PNNs surrounding DCN neurons is due to a shift in the balance between synthetic and catabolic mechanisms in favor of the latter. PNNs in the DCN are composed of CSPGs, tenascin-R, hyaluronic acid and the link proteins cartilage LP1 and Bral2. Most of these substances are produced by both neurons and glia, but only PNN-bearing nerve cells express cartilage LP1 and HASs [19]. These are crucial elements for the formation and maintenance of the net. Cartilage LP1 is essential for PNN formation, likely allowing the capture of CSPGs into the hyaluronan perineuronal coat [11]. Hyaluronan is a component of all organized matrices, binding to all the lecticans and attaching the matrix to the cell surface [37]. DCN neurons also express MMP2 and MMP9 ([27] and this study). These enzymes are upregulated and activated following enhanced neuronal activity and synaptic transmission [38]–[40], and are involved in activity-dependent structural remodeling [39], [41]. Most importantly, several PNN components are MMP targets [26], [42]. Therefore, DCN neurons are equipped with both the molecular machinery needed to assembly ECM structures on their surface, and the degrading enzymes that can be used to modulate PNN thickness and composition.

EE acted synergistically on both mechanisms. It reduced the content of mRNA for PNN key molecules in the DCN neurons, in line with recent studies reporting activity-dependent changes in the neuron transcription machinery that may be related to plasticity [14], [21], [43]. At the same time, EE increased the enzymatic activity of MMP2 and MMP9 in nuclear neurons. Indeed, although ISZ staining did not allow us to detect gelatinolytic activity in extracellular spaces, we were able to demonstrate a clear-cut relationship between intracellular ISZ labeling and PNN intensity in individual neurons, which is fully consistent with local regulation of ECM composition. The direct link between changes in PNN structure and neuritic plasticity was further corroborated by the phenotype of cartilage LP1 mutant mice, in which impaired PNN assembly is associated with oversized PC terminals, even in the absence of external stimulation. Most interestingly, however, our observations indicate that, although most of the changes occur in the postsynaptic DCN neurons, regulation of PNN maintenance and structure is also dependent on presynaptic partners, and notably PC axons. The intrinsic growth properties of PCs influenced growth-control mechanisms in basal conditions, and considerably amplified the effects produced by EE. Furthermore, ablation of PC boutons induced a dramatic attenuation of PNNs. Together, these observations suggest that PNN intensity is related to the strength of PC inhibitory input to DCN neurons: conditions that reduce this influence shift the balance of regulatory mechanisms towards the reduction of PNN thickness. In line with this view, in the visual cortex, block of glutamatergic transmission has no effect [44], whereas decreased GABAergic activity causes PNN attenuation [45]. This is consistent with a general mechanism in which the level of control on plastic processes is set by the concomitant strength of inhibition [46], that can be modified by exposure to EE [20]. In this scenario, our observations indicate that PC axons modulate the balance between synthesis and degradation of PNN components in the postsynaptic DCN neurons. In addition, the changes of ISZ labeling induced by EE in PCs suggest that the presynaptic axons may actively contribute to the removal of ECM components.

### EE-dependent changes are influenced by intrinsic properties of PCs

An interesting finding of our experiments is that both neuritic plasticity and PNN regulation are strongly influenced by the intrinsic growth properties of PCs. Selective overexpression of GAP-43 in these neurons induces strong growth-promoting effects, which become particularly evident following axotomy [3]. The injured axons of transgenic PCs elongate into non-permissive territories and have reduced sensitivity to extrinsic growth-inhibitory molecules [47], [48]. The present observations highlight novel features of intact L7/GAP-43 PCs, whose terminal boutons in the DCN are smaller than their wild-type counterparts. The consequences of this morphological phenotype on the function of the synapse between PCs and DCN neurons are not clear. However, they do not interfere with the quality of the structural modifications set up in response to environmental stimulation.

Already in ST conditions, transgenic cerebella are characterized by expression levels of mRNAs for PNN components or levels of activity of ECM degrading enzymes that are comparable to those observed in wild-type animals exposed to EE. In addition, all the effects of EE on PC neurites and PNNs are significantly amplified in transgenic mice. Thus, enhanced intrinsic growth properties of the presynaptic PCs lower the levels of growth-inhibitory control and, consequently, a more robust response to environmental stimulation is developed. In line with the well-established role of GAP-43 in synaptic plasticity [49], these findings indicate that the activity of neuronal growth-associated genes is not limited to strengthen the intrinsic molecular machinery that sustains neuritic elongation, but is also efficient in making the surrounding milieu permissive for process outgrowth and structural remodeling. Such transynaptic effects are likely mediated via GAP-43-operated modulation of synaptic transmission [50].

The comparison of wild-type and transgenic mice exposed to EE shows that overexpression of neuronal growth genes and external stimuli act synergistically to boost neuritic growth while reducing PNN-derived extrinsic inhibition. In this context, the balance between intrinsic neuronal properties and extrinsic cues plays an essentially permissive role by setting the propensity of neural circuits to modify their structure in response to external stimuli. The latter exert a primarily instructive action to determine the actual shape of the newly-formed connections [1]. In addition, however, environmental stimulation directly modulates local regulatory mechanisms in order to facilitate the remodeling of neuronal connectivity. This effect is likely operated through specific patterns of neuronal activity and synaptic transmission induced by the interaction with the external world, which are restricted to the neural structures directly involved in the experience-dependent event. In this way, specific external stimuli can transiently dampen growth-inhibitory mechanism along defined neural circuits so to induce appropriate structural remodeling and meaningful functional adaptation, while preventing unwanted growth and maladaptive outcomes.

## Materials and Methods

### Animals

Experiments were performed on 3-month-old FVB female wild-type mice (Harlan Italy), and transgenic mice overexpressing GAP-43 under the Purkinje cell-specific promoter L7 [3], generated by Dr Joost Verhaagen (Netherlands Institute for Neuroscience, Amsterdam, The Netherlands). In addition, mutant Balb-c mice lacking expression of cartilage LP1 [23] in the adult CNS and wild-type mice of the same strain were examined. In a few instances, we used Pax2-GFP transgenic mice to visualize GABAergic interneurons [51]. The number of animals used in the different experiments is reported in **Table S1**.

All surgical procedures were performed under deep general anesthesia obtained by intraperitoneal administration of ketamine (100 mg/kg; Ketavet) supplemented by xylazine (5 mg/kg; Rompun). The experimental plan was designed according to the European Communities Council Directive of 1986 (86/609/EEC), National Institutes of Health guidelines and the Italian law for care and use of experimental animals (DL116/92) and was approved by the Italian Ministry of Health and by the Bioethic Committee of the University of Turin.

### Experimental paradigms and surgery

For EE paradigm, 15–20 3-month-old mice were housed for one month in a large cage (78×48×82 cm) containing toys, tubes and running wheels. Disposition of tools, food and water was changed daily to achieve higher levels of stimulation and associated physical activity. In ST conditions, groups of 5 mice were housed in conventional cages (42.5×27×18 cm).

Selective degeneration of PCs in discrete cortical regions, and consequent denervation of DCN neurons (**Fig. 7SA,D**), was induced by intraparenchymal injection of 1 µl of propidium iodide (1 mg/ml in sterile saline solution), as previously described [29]. Briefly, anaesthetized mice were placed in a stereotaxic apparatus and a small hole was drilled in the occipital bone to gain access to the vermis. Propidium iodide was pressure injected by a glass capillary connected to a PV800 Pneumatic Picopump (WPI, New Haven, CT). The animals were killed 14 days after injection.

### Histological procedures

Animals were anaesthetized and transcardially perfused with 200 ml of 4% paraformaldehyde in 0.12 M phosphate buffer. Brains were dissected and postfixed overnight at 4°C, then cryoprotected in 0.12 M phosphate buffer containing 30% sucrose at 4°C, until they sank. Cerebella were cut on a cryostat into 25-µm-thick sagittal sections and collected in phosphate-buffered saline (PBS).

Primary antibodies (see **Table S2** for details) were incubated overnight at room temperature (RT) in PBS containing 0.25% Triton X-100, 0.1% sodium azide and 1.5% serum of the species of the second antibody: mouse SMI32, to stain DCN projection neurons; mouse/rabbit anti-calbindin D28k, to stain PCs; rabbit anti-v-glut2, to stain glutamatergic terminals; rabbit anti-VGAT, to stain GABAergic terminals; mouse anti-S-100β, to stain astrocytes. To visualize PNNs, sections were incubated in biotinylated *Wisteria floribunda* agglutinin (WFA) or biotinylated hyaluronan binding protein (HABP) for 2 h at RT. Subsequently, sections were incubated for 1 h at RT with one of the following secondary antibodies conjugated to fluorophores: biotinylated goat anti-rabbit (Vector Laboratories), streptavidin Texas Red (Vector), horse anti-mouse AMCA (Vector), donkey anti-mouse or anti-rabbit Alexa Fluor 488 (Invitrogen), donkey anti-mouse Alexa Fluor 546 (Invitrogen). To stain MMPs, slices were preincubated 30 min with sodium borohydride 1% in Tris Buffered Saline (TBS), and incubated overnight at RT with rabbit anti-MMP2 or for 48 h at 4°C with rabbit anti-MMP9, with 1.5% normal serum of the species of the second antibody. Sections were processed for immunofluorescence with the Tyramide Signal Amplification Biotin System (PerkinElmer) following the manufacturer's protocol, and subsequently revealed by 40 min incubation at RT with streptavidin Texas Red. For each immunohistochemical reaction, slices from all experimental conditions were processed together and incubation times kept constant. Finally, the sections were mounted in Mowiol (Calbiochem).

Histological preparations were examined under a Zeiss Axiophot light microscope or a Leica SP5 confocal microscope (Leica Microsystems). Confocal images were taken at a resolution of 512×512 dpi and 100 Hz speed and each focal plane was 1-µm-thick. Lasers intensity, gain and offset were maintained constant in each analysis. Quantitative and morphometric evaluations were made using the software Image J (Research Service Branch) and Neurolucida (MicroBrightField), the latter connected to an E-800 Nikon microscope via a color CCD camera.

### Quantification of size and number of PC terminals

The DCN comprise projection neurons and local interneurons. Our analysis was focused on the former neurons of the medial DCN, which can be selectively identified by their size and SMI32 antibody staining [52], and bear prominent WFA-positive PNNs [19]. The size of PC terminals and their number/mm of postsynaptic membrane length were evaluated by means of ImageJ software on 1-µm-thick confocal images (1 focal plane), captured under a 63x objective. The size was evaluated by drawing the outline of PC terminals, visualized by anti-calbindin staining (80–320 terminals/mouse, N = 5 mice/experimental condition). Since anti-calbindin antibodies stain the entire PC, the identification of terminal boutons was confirmed by additional staining with anti-VGAT (vesicular GABA transporter) antibodies. All measurements were performed on DCN neurons in which the nucleus was visible in the optical section. To study the combined effect of the environmental stimulation and GAP-43 overexpression, we compared the change in dimensions after EE in wild-type and transgenic animals by calculating the ratio between the measurements of button size obtained in ST and EE conditions in each strain. The measures of number of terminals/mm of postsynaptic membrane were corrected by applying the Abercrombie formula [53] to compensate for the size changes of terminal boutons in different experimental conditions.

### Evaluation of glutamatergic terminal density

To estimate the density of v-glut2-positive glutamatergic axon terminals, we selected four sections containing the medial DCN for each animal (N = 4 for wild-type ST and EE mice; N = 7 for transgenic ST and EE mice), and captured two 1-µm-thick confocal images/section under a 63x objective. On such images, we used the “analyze particle” function of ImageJ to estimate the density of boutons (number of terminals/mm^2^).

### Measurements of dimensions of PC and DCN neuron somata

The perikaryal size of PCs and DCN projection neurons was determined using ImageJ on 1-µm-thick confocal images captured under a 63x objective. For each animal, we measured the area of about 40 PCs (visualized with anti-calbindin antibodies; N = 3 mice/experimental condition) and about 10 DCN neurons (stained by SMI32 antibodies; N = 4 wild-type ST, 10 wild-type EE, 4 transgenic ST, 9 transgenic EE), in which the nucleus was visible.

### Quantification of PNNs

The number of SMI32-positive projection neurons bearing a PNN was determined on double labeled sections with SMI32 antibodies and WFA histochemistry. In each section (n = 4), we sampled all the SMI32-positive DCN neurons and assessed the presence of a PNN surrounding each individual neuron (N = 5 mice/experimental condition).

Analysis of WFA or HABP staining intensity was performed on confocal images collected under a 63× objective. By ImageJ, we measured the brightness intensity (range 0–255) of at least 30 PNNs/animal (N = 5 mice/experimental condition) by randomly selecting 15 pixels in the net and calculating their average. The background brightness, taken from a non-stained region of the cortical molecular layer, was subtracted from the brightness measurements. Each net was assigned to one of three categories of staining intensity, ranging from the lowest to the highest value of WFA/HABP intensity: faint = 0–33%, medium = 34–66%, strong = 67–100% of maximum staining intensity.

### Real-time PCR

Mice were anaesthetized and decapitated. Cerebella were rapidly removed and cut in frontal sections using a vibratome. Under a dissection microscope, the DCN were isolated and DCN of two mice of the same experimental condition were pooled together. RNA from DCN pools was extracted using the RNeasy Micro kit (Qiagen) and retrotranscribed using a cDNA synthesis kit (Applied Biosystems).

Real Time PCR experiments were conducted using the ABI Prism 7000 Sequence Detection System instrumentation in combination with Taqman reagent-based chemistry (all from Applied Biosystems). PCR amplifications were run on 3 pools/experimental condition in triplicate, together with water or negative retro-transcription reactions; for the PCR conditions see [54]. β2-microglobulin was used as the reference gene. We collected data from at least three different experiments and we analyzed them using the 7000 v1.1 SDS instrument software (Applied Biosystems). Relative quantifications were obtained using the 2-ΔΔct method [55]. Resultsfrom the EE or the transgenic condition were expressed as fold increase/decrease relative to the standard or the WT condition, respectively.

### *In Situ* Hybridization

To synthesize digoxigenin (dig)-labeled RNA probes, a first-strand cDNA was synthesized from 2 µg of mouse brain RNA using a cDNA synthesis kit from Applied Biosystems. The PCR product was amplified using a 5′ primer containing a T7 phage promoter sequence (Sigma) and a 3′ primer containing a SP6 phage promoter sequence (Sigma), generating a template for transcription of a sense and an antisense probe, respectively. The *in vitro* transcription reaction was performed using dig-UTP RNA labelling mix (Roche) and SP6 or T7 RNA polymerase (Roche) following the manufacturer's instructions. The size of each probe corresponded to that of the specific PCR product. For the primer sequences, the size of the PCR products and the specific cDNA region amplified see [19].

*In situ* hybridization for cartilage LP1, aggrecan HAS2 was performed on coronal sections of adult mouse cerebellum. Mice were decapitated and the cerebellum was removed. The tissue block was covered with OCT embedding medium and rapidly frozen at −40°C in 2-methylbutane. Sections 14-µm-thick were cut on a cryostat, collected on Superfrost slides (Thermo Scientific), and air-dried at RT. Then sections were fixed in 4% paraformaldehyde for 10 min, permeabilized for 10 min in PBS with 0.5% Triton X-100 and acetylated by 10 min incubation in a solution made of 250 ml of water with 3.5 ml of triethanolamine and 625 µl of acetic anhydride added dropwise. Prehybridization was performed in hybridization buffer (50% formamide, 5x SSC, 2% blocking reagent; Roche) for 3 h RT. Hybridization with dig-labelled probes (100 ng/ml) was performed in the same buffer overnight at 69°C. Stringency washing was done in 0.2x SSC for 1 h at 69°C. For the detection of dig-labelled hybrids the slides were incubated for 1 h RT with 1% blocking reagent made in maleic acid buffer (blocking buffer), and then for 1 h with alkaline phosphatase-conjugated anti-dig antibodies (Roche) 1:5000 in blocking buffer. The slides were washed twice for 30 min in maleic acid buffer and incubated overnight in colour development buffer (2.4 mg levamisole, 45 µl 4-nitroblue tetrazolium and 35 µl 5-bromo-4-chloro-3-indolyl-phosphate, all from Sigma, in 10 ml of a buffer made of 0.1 M Trizma base, 0.1 M NaCl and 0.005 M MgCl_2_, pH 9.5). The colour development reaction was stopped in neutralizing buffer (0.01 M Trizma base and 0.001 M EDTA, pH 8). The sections were mounted in Mowiol and coverslipped.

### Evaluation of MMP2 and MMP9 staining intensity

The outline of MMP2-positive or MMP9-positive cell bodies of DCN neurons was drawn by means of Neurolucida software under a 40x objective and the brightness of immunofluorescence (range 0-255) was measured (50 cells/animal, N = 3 mice/condition). The background brightness, taken from a non-stained region of the cerebellar white matter, was subtracted from the brightness measurement.

### MMPs activity

High resolution ISZ [28] was used to evaluate MMPs enzymatic activity. Mice were anaesthetized and decapitated. Cerebella were removed and stored in a solution of methanol and ethanol plus 5% polyvinylpirrolidone overnight at 4°C. Subsequently, cerebella were washed in ethanol plus polyvinylpirrolidone and immersed in mixtures of ethanol and polyester wax (Science Services) with increasing concentration of wax and raising the temperature up to 42°C. Embedded specimens were cut into 6-µm-thick sections using a rotary microtome equipped with the running water path and mounted on Superfrost slides. Sections were dewaxed in pure ethanol at 37°C, rinsed and preincubated in ultrapure water at 37°C for 1 h, then covered with a fluorogenic substrate DQ gelatin (Invitrogen) 1:100 in the buffer supplied by the manufacturer and incubated at 37°C for 2 h. Subsequently, sections were postfixed in 4% paraformaldehyde in 0.12 M phosphate buffer for 15 min at RT and rinsed in PBS. Slides were counterstained with WFA-bio, anti-calbindin or DAPI, then revealed with 1 h incubation in streptavidin Alexa Fluor 555 and donkey anti-rabbit Alexa Fluor 647 in PBS and 5% donkey serum. All incubation times were maintained constant. Negative controls were obtained by processing some slices with the specific MMP inhibitor phenanthroline (50 mM, Invitrogen) and counterstaining them with DAPI. Dry slides were coverslipped using Vectashield (Vector).

MMPs activity was evaluated in PCs and in the DCN neurons (N = 4 wild-type ST, 6 wild-type EE; N = 4 transgenic ST, 8 transgenic EE). ISZ signal was evaluated by measuring the intensity of the fluorescent signal as described above for MMP immunocytochemistry. The percentage of calbindin-positive PCs (of the IV-V lobules) that were ISZ-positive was calculated (three slices/animal). Moreover, by ImageJ software we evaluated the ISZ intensity in 8–27 PCs/animal of the lobule IV-V of the cerebellum on confocal images acquired under a 40x objective, by selecting the cell body of the calbindin-positive cells and then evaluating the ISZ intensity signal. After subtracting the ISZ intensity of the molecular layer (background value, because it was a non-stained region) from each value, we classified each ISZ value as faint, medium or strong according to their brightness intensity, ranging from the lowest to the highest value of ISZ intensity of PCs.

To investigate the relationship between gelatinase activity and PNN structure, for each DCN neuron sampled we measured the intensity of ISZ reaction in the cell body and of WFA labeling of the surrounding net. The obtained data were plotted and linear regression equations were fitted for each animal and for each experimental condition. In addition, to compare the angular coefficients of the regression lines obtained in the different conditions, the regression equations were plotted by imposing the same intercept value.

### Statistical analyses

In all cases, the experimenters were blinded to the manipulations. Data elaboration and statistical analyses were conducted by means of SigmaStat 3.5. In all instances, we set the value of α = 0.05 and *P*<0.05 was considered as statistically significant; statistical parameters are reported in the figure legends. Graphs and data are represented as mean ± standard error of the mean (SEM) unless specified. If data sets passed normality test and equal variance test, we performed Student's t-test or One Way Anova followed by Bonferroni *post hoc* tests. To compare different percentages, we transformed the values in radiants according to the arcsin transformation. We used χ^2^-test to compare the distribution of frequencies relative to staining intensity categories. To study the regression equations, we performed normality test, constant variance test and analysis of variance, then the angular coefficients of wild-type and transgenic mice were compared by One Way Anova.

## Supporting Information

Figure S1 Perikaryal size of PCs and DCN neurons following EE. (A-D) examples of PC perikarya visualized by anti-calbindin immunocytochemistry in the different experimental conditions. (E) Quantification of PC somatic size (One Way Anova; N  =  3 mice/experimental condition). (F-I) DCN neurons labeled by SMI32 antibodies. (J) Quantification of the perikaryal size of these neurons (One Way Anova; N  =  4 wild-type ST, 10 wild-type EE; 4 transgenic ST, 9 transgenic EE). Scale bar: 10 μm. WT: wild-type; TG: transgenic; ST: standard; EE: enriched; CaBP: calbindin; SMI32: neurofilament-H non-phosphorylated.
(TIF)

Figure S2 Analysis of PNN bearing neurons in individual cerebellar nuclei. The histograms illustrate the percentage of SMI32-positive neurons bearing a WFA-positive net in the medial (A), interpositus (B) and lateral (C) DCN nucleus (in all cases: One Way Anova; N  =  5 mice/experimental condition). **P < 0.01.
(TIF)

Figure S3 Expression of mRNA coding for PNN molecules in the mouse DCN. *In situ* hybridization showed the expression of cartilage link protein-1 (Crtl1; A) and aggrecan (B) mRNAs in DCN neurons. Scale bar: 40 μm.
(TIF)

Figure S4 MMP9 expression in the cerebellum. In the adult mouse cerebellum MMP9 (red) is expressed by PCs (anti-calbindin, green in A-A_2_), DCN projection neurons, (SMI32, blue, B-B_2_) and interneurons (Pax2-GFP mice, green; C-C_2_). MMP9 is also expressed by glial cells, as seen with anti-S100β abs (blue; D-D_2_). Scale bars: 20 μm. CaBP: calbindin; SMI32: neurofilament-H non-phosphorylated; GFP: green fluorescent protein; S100β: S100 calcium binding protein β.
(TIF)

Figure S5 MMP activity in PCs of WT and L7/GAP-43 mice after EE. (A-D_1_) PCs, stained by anti-calbindin antibodies (blue), show MMP activity, revealed by ISZ (green). (E) Percentage of PCs that show ISZ signal (One Way Anova; N  =  4 wild-type ST, 6 wild-type EE, 4 transgenic ST, 8 transgenic EE). (F) Analysis of the fluorescence intensity of the ISZ signal in PCs. Scale bars: 20 μm, 10 μm in the insets (χ^2^-test: 87.67 with 3 DF). WT: wild-type; TG: transgenic; ST: standard; EE: enriched; CaBP: calbindin; ISZ: *in situ* zymography.
(TIF)

Figure S6 MMP activity is inhibited by phenanthroline. (A,B) Control slices prepared for ISZ were incubated with the general MMP inhibitor phenanthroline at a concentration of 50 mM. (A,A_1_) In the cerebellar cortex, neither PCs nor other cell types (blue) showed ISZ signal (green) after treatment with the inhibitor. Similarly, in the DCN (B,B_1_) the incubation with phenanthroline completely abolished the ISZ signal (green). (A_1_,B_1_) The diffused fluorescence shown in negative control slices is similar to the ISZ background level we measured in the molecular layer. The blue color is DAPI staining. Scale bar: 50 μm. ISZ: *in situ* zymography; DAPI: 4′,6-diamidino-2-phenylindole.
(TIF)

Figure S7 Selective PC degeneration induced by propidium iodide injections. (A) shows the pattern of PC degeneration highlighted by anti-calbindin immunostaining (red, asterisk points to the approximate position of the propidium iodide injection site). (B) shows the same section as seen in the green channel showing GFP labeling highlighting GABAergic interneurons: note the selective effect of propidium iodide on PCs. (C,D) Higher magnification pictures showing the distribution pattern of calbindin-immunolabeled PC terminals in intact (C) and partially denervated nuclei (D; 14 days after propidium iodide injection). Scale bars: 500 μm in A and B, 100 μm in C and D. CaBP: calbindin.
(TIF)

Table S1 Number of mice used in each experiment. ST: standard; EE: enriched; TG: transgenic; PI: propidium iodide injected; Crtl1: cartilage link protein-1; KO: knockout; IHC: immunohistochemistry; ISH: *in situ* hybridization; ISZ: *in situ* zymography; PCR: real-time polymerase chain reaction.
(DOC)

Table S2 Primary antibodies and markers used in our experiments.
(DOC)
