# Supplementary figures and images for: Experience-Dependent Plasticity and Modulation of Growth Regulatory Molecules at Central Synapses
Source: PLoS One. 2011 Jan 31;6(1):e16666. doi: 10.1371/journal.pone.0016666 (PMC3031615; doi:10.1371/journal.pone.0016666)

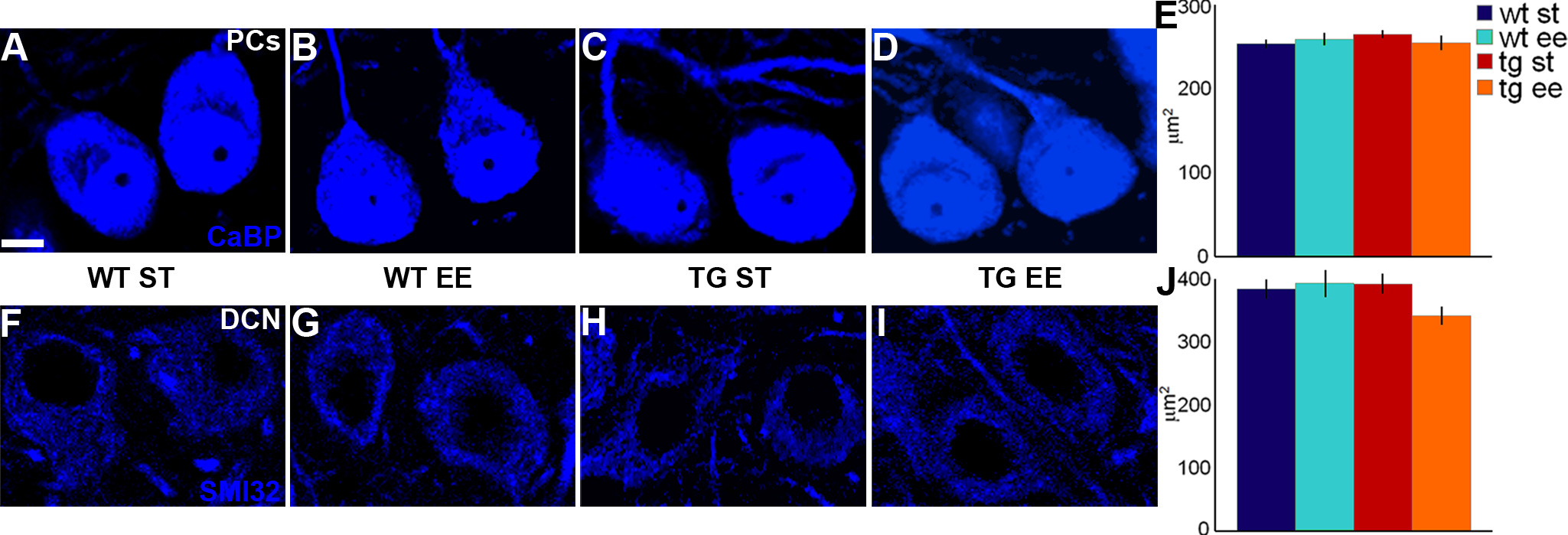

Supplement: Figure S1 — Perikaryal size of PCs and DCN neurons following EE. (A-D) examples of PC perikarya visualized by anti-calbindin immunocytochemistry in the different experimental conditions. (E) Quantification of PC somatic size (One Way Anova; N = 3 mice/experimental condition). (F-I) DCN neurons labeled by SMI32 antibodies. (J) Quantification of the perikaryal size of these neurons (One Way Anova; N = 4 wild-type ST, 10 wild-type EE; 4 transgenic ST, 9 transgenic EE). Scale bar: 10 μm. WT: wild-type; TG: transgenic; ST: standard; EE: enriched; CaBP: calbindin; SMI32: neurofilament-H non-phosphorylated. (TIF) [file pone.0016666.s001.tif]

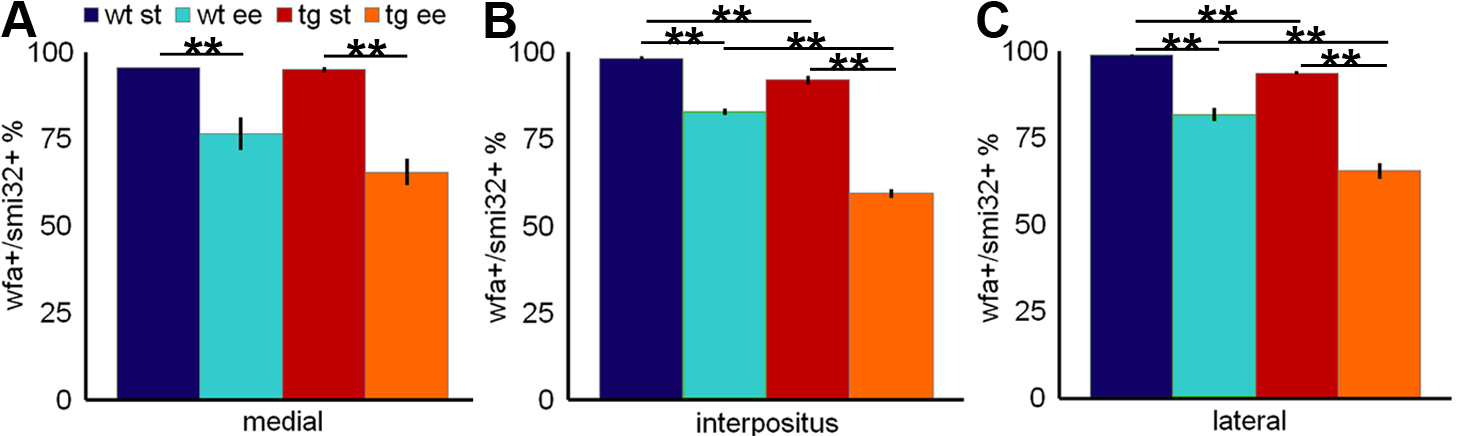

Supplement: Figure S2 — Analysis of PNN bearing neurons in individual cerebellar nuclei. The histograms illustrate the percentage of SMI32-positive neurons bearing a WFA-positive net in the medial (A), interpositus (B) and lateral (C) DCN nucleus (in all cases: One Way Anova; N = 5 mice/experimental condition). **P < 0.01. (TIF) [file pone.0016666.s002.tif]

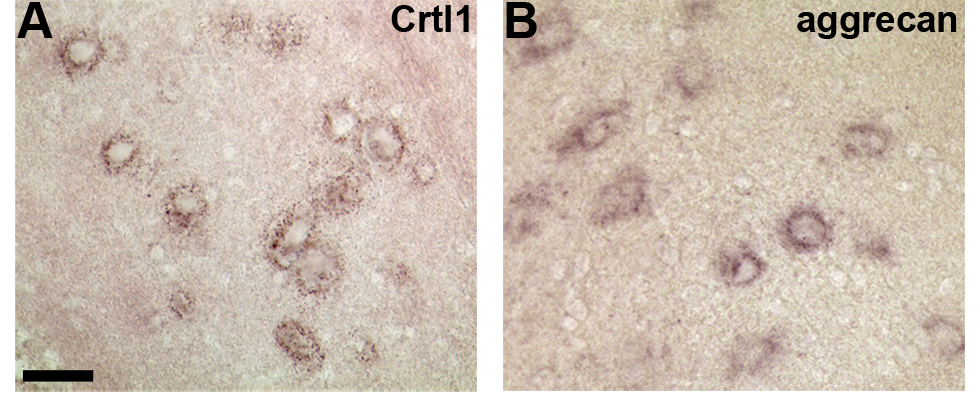

Supplement: Figure S3 — Expression of mRNA coding for PNN molecules in the mouse DCN. In situ hybridization showed the expression of cartilage link protein-1 (Crtl1; A) and aggrecan (B) mRNAs in DCN neurons. Scale bar: 40 μm. (TIF) [file pone.0016666.s003.tif]

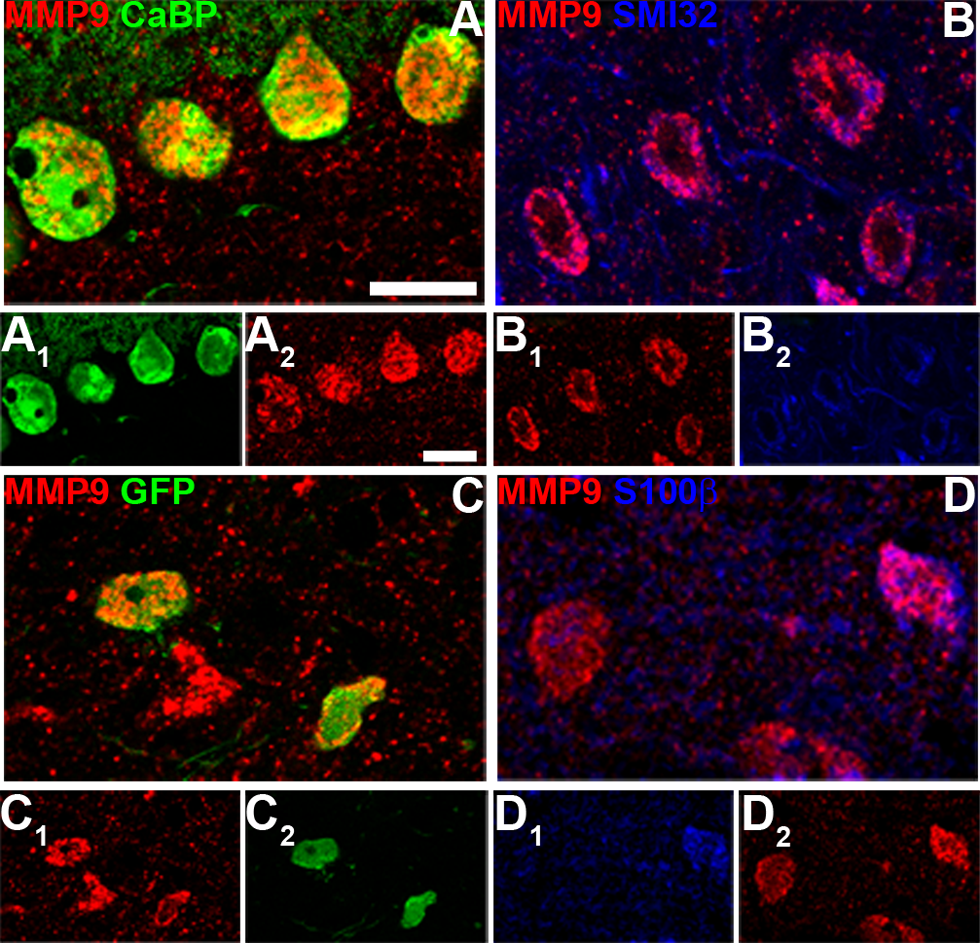

Supplement: Figure S4 — MMP9 expression in the cerebellum. In the adult mouse cerebellum MMP9 (red) is expressed by PCs (anti-calbindin, green in A-A2), DCN projection neurons, (SMI32, blue, B-B2) and interneurons (Pax2-GFP mice, green; C-C2). MMP9 is also expressed by glial cells, as seen with anti-S100β abs (blue; D-D2). Scale bars: 20 μm. CaBP: calbindin; SMI32: neurofilament-H non-phosphorylated; GFP: green fluorescent protein; S100β: S100 calcium binding protein β. (TIF) [file pone.0016666.s004.tif]

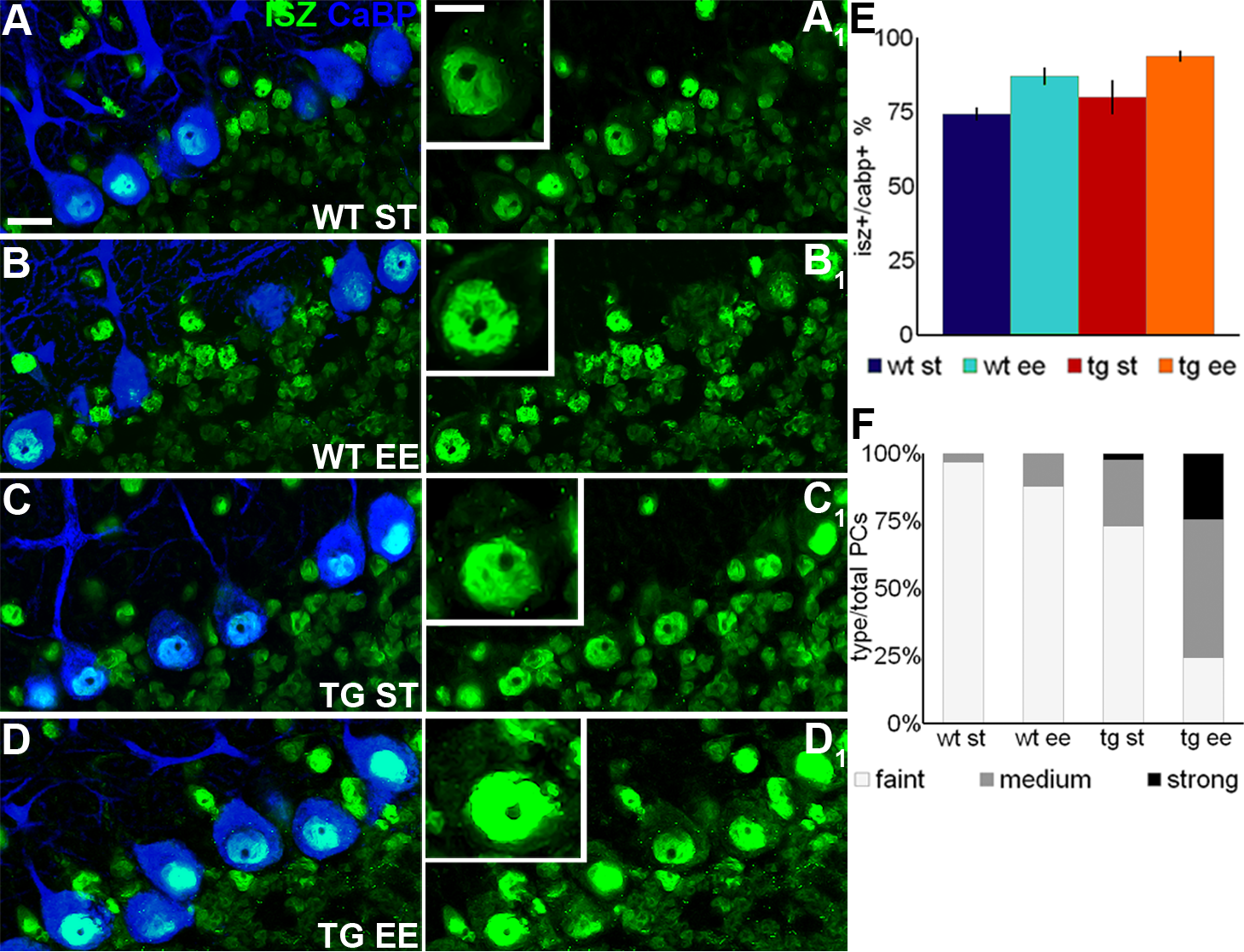

Supplement: Figure S5 — MMP activity in PCs of WT and L7/GAP-43 mice after EE. (A-D1) PCs, stained by anti-calbindin antibodies (blue), show MMP activity, revealed by ISZ (green). (E) Percentage of PCs that show ISZ signal (One Way Anova; N = 4 wild-type ST, 6 wild-type EE, 4 transgenic ST, 8 transgenic EE). (F) Analysis of the fluorescence intensity of the ISZ signal in PCs. Scale bars: 20 μm, 10 μm in the insets (χ2-test: 87.67 with 3 DF). WT: wild-type; TG: transgenic; ST: standard; EE: enriched; CaBP: calbindin; ISZ: in situ zymography. (TIF) [file pone.0016666.s005.tif]

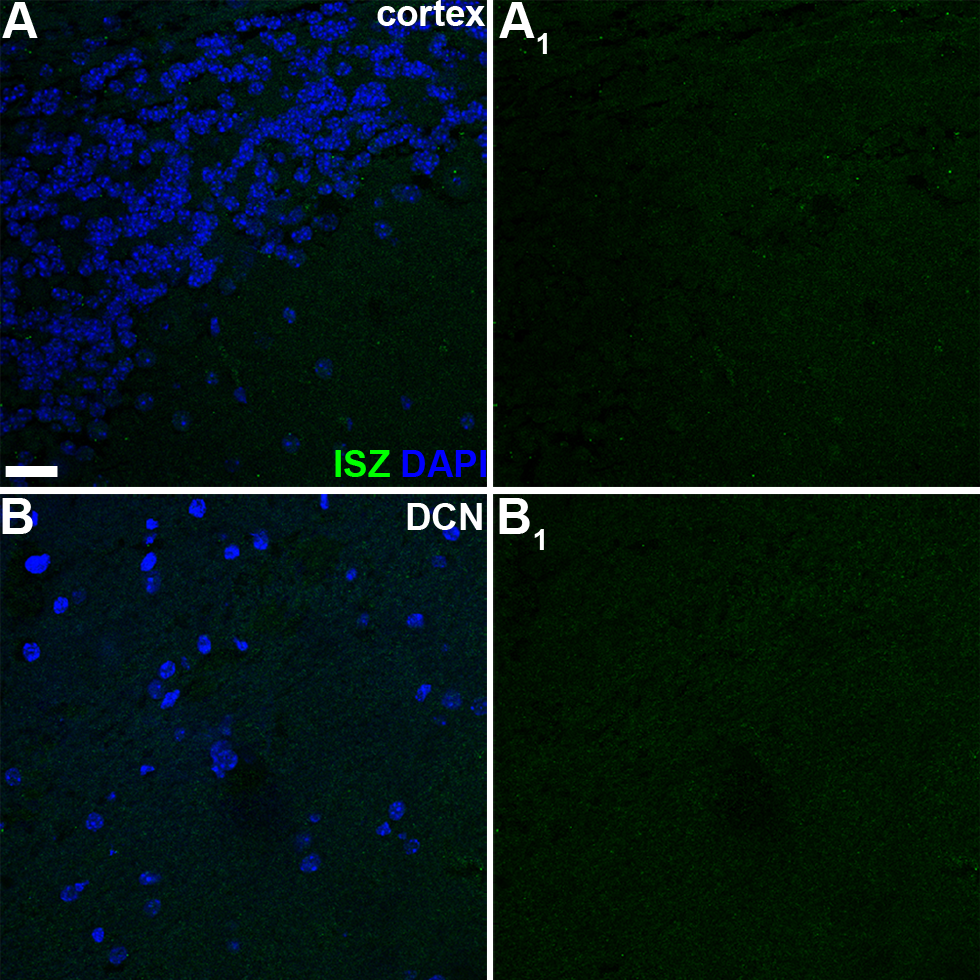

Supplement: Figure S6 — MMP activity is inhibited by phenanthroline. (A,B) Control slices prepared for ISZ were incubated with the general MMP inhibitor phenanthroline at a concentration of 50 mM. (A,A1) In the cerebellar cortex, neither PCs nor other cell types (blue) showed ISZ signal (green) after treatment with the inhibitor. Similarly, in the DCN (B,B1) the incubation with phenanthroline completely abolished the ISZ signal (green). (A1,B1) The diffused fluorescence shown in negative control slices is similar to the ISZ background level we measured in the molecular layer. The blue color is DAPI staining. Scale bar: 50 μm. ISZ: in situ zymography; DAPI: 4′,6-diamidino-2-phenylindole. (TIF) [file pone.0016666.s006.tif]

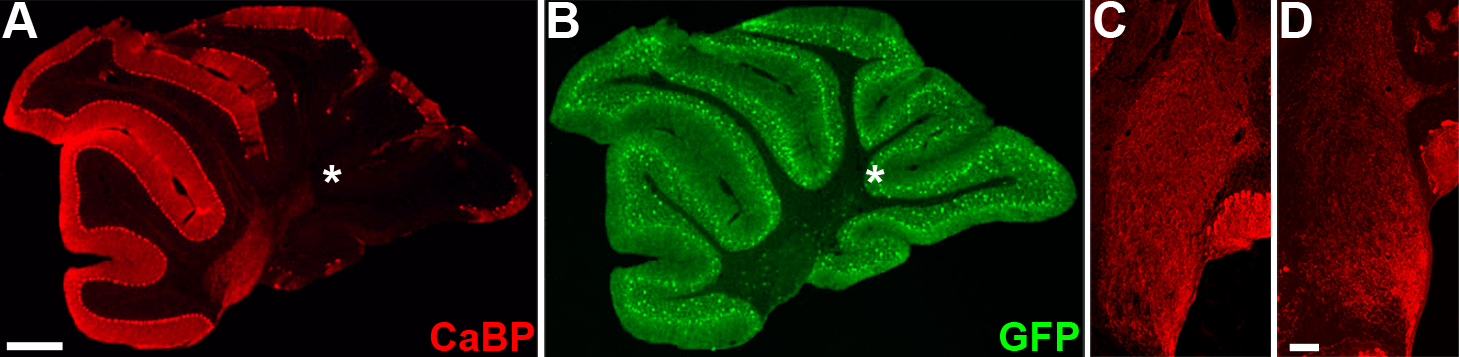

Supplement: Figure S7 — Selective PC degeneration induced by propidium iodide injections. (A) shows the pattern of PC degeneration highlighted by anti-calbindin immunostaining (red, asterisk points to the approximate position of the propidium iodide injection site). (B) shows the same section as seen in the green channel showing GFP labeling highlighting GABAergic interneurons: note the selective effect of propidium iodide on PCs. (C,D) Higher magnification pictures showing the distribution pattern of calbindin-immunolabeled PC terminals in intact (C) and partially denervated nuclei (D; 14 days after propidium iodide injection). Scale bars: 500 μm in A and B, 100 μm in C and D. CaBP: calbindin. (TIF) [file pone.0016666.s007.tif]
