# Supplementary material for: Experience-Dependent Plasticity and Modulation of Growth Regulatory Molecules at Central Synapses
Source: PLoS One. 2011 Jan 31;6(1):e16666. doi: 10.1371/journal.pone.0016666 (PMC3031615; doi:10.1371/journal.pone.0016666)
Supplement: Table S1 — Number of mice used in each experiment. ST: standard; EE: enriched; TG: transgenic; PI: propidium iodide injected; Crtl1: cartilage link protein-1; KO: knockout; IHC: immunohistochemistry; ISH: in situ hybridization; ISZ: in situ zymography; PCR: real-time polymerase chain reaction. (DOC) [file pone.0016666.s008.doc]

	FVB ST	FVB
EE	GAP-43 TG ST	GAP-43 TG EE	FVB 
PI 14d	GAP-43 TG PI 14d	Balb-c	Crtl1
KO	Pax2-GFP 	
IHC	5	10	7	9	4	4	3	4	2	
ISH	3									
PCR	18	18	18	18						
ISZ	4	6	4	8						
Total	30	34	29	35	4	4	3	4	2	
