# Supplementary material for: Experience-Dependent Plasticity and Modulation of Growth Regulatory Molecules at Central Synapses
Source: PLoS One. 2011 Jan 31;6(1):e16666. doi: 10.1371/journal.pone.0016666 (PMC3031615; doi:10.1371/journal.pone.0016666)
Supplement: Table S2 — Primary antibodies and markers used in our experiments. (DOC) [file pone.0016666.s009.doc]

Primary antibodies	Species of origin	Working dilutions	Supplier and catalogue no.	
anti-Calbindin D28k	Mouse	1:1500	Swant, Swiss Antibodies, Bellinzona, Switzerland. #300	
anti-Calbindin D28k	Rabbit 	1:1500	Swant, Swiss Antibodies, Bellinzona, Switzerland. #CB38	
DAPI		1:500	Fluka/Sigma Aldrich 32670	
anti-GFP	Rabbit	1:700	Invitrogen/Molecular Probes, Eugene, OR, USA. #A-11122	
anti-MMP2	Rabbit	1:2000	Abcam, Cambridge, UK. #ab37150	
anti-MMP9	Rabbit	1:5000	Chemicon/Millipore, Billerica, MA, USA. #AB16996	
anti-S100b	Mouse	1:500	Sigma Aldrich, Italy. #S2532	
SMI-32	Mouse	1:500	Sternberger/Covance Europe. #SMI-32R	
anti-v-glut2	Rabbit	1:500	Synaptic System, Göettingen, Germany. #135402	
anti-VGAT	Rabbit	1:4000	gift from Dr M. Sassoé-Pognetto, University of Turin, Italy	
WFA-bio		1:200 – 20mg/ml	Sigma Aldrich, Italy. #L-1516	
HABP-bio		1:100 –
10mg/ml	Seikagaku, Falmouth, MA. #400763-1A	
